# The genome sequence of a lichen-forming fungus, *Platismatia glauca *Linnaeus, 1753

**DOI:** 10.12688/wellcomeopenres.22842.1

**Published:** 2024-08-12

**Authors:** Rebecca Yahr, Royal Botanic Garden Edinburgh Genome Acquisition Lab, Darwin Tree of Life Barcoding collective, Wellcome Sanger Institute Tree of Life Management, Samples and Laboratory team, Wellcome Sanger Institute Scientific Operations: Sequencing Operations, Wellcome Sanger Institute Tree of Life Core Informatics team, Tree of Life Core Informatics collective, Darwin Tree of Life Consortium

**Affiliations:** 1 Royal Botanic Garden Edinburgh, Edinburgh, Scotland, UK

**Keywords:** Platismatia glauca, lichen-forming fungus, genome sequence, chromosomal, Lecanorales

## Abstract

We present a genome assembly from a specimen of *Platismatia glauca* (lichen-forming fungus; Ascomycota; Lecanoromycetes; Lecanorales; Parmeliaceae). The genome sequence is 33.2 megabases in span. Most of the assembly is scaffolded into 21 chromosomal pseudomolecules. The mitochondrial genome has also been assembled and is 95.06 kilobases in length.

## Species taxonomy

Eukaryota; Opisthokonta; Fungi; Dikarya; Ascomycota; saccharomyceta; Pezizomycotina; leotiomyceta; Lecanoromycetes; OSLEUM clade; Lecanoromycetidae; Lecanorales; Lecanorineae; Parmeliaceae; *Platismatia*; *Platismatia glauca* Linnaeus, 1753 (NCBI:txid78070).

## Background

*Platismatia glauca* is a member of one of the largest and most variable families of fungi that form lichens – the *Parmeliaceae*, a family which displays a remarkable diversity of shapes and sizes, from long pendant hairlike forms, hundreds of leafy species and even inconspicuous crusts and parasitic fungi that have lost the ability to form lichens ( Divakar *et al.*, 2015). *Platismatia glauca* itself is a large and conspicuous leafy (foliose) lichen, widely distributed and abundant across the northern temperate and boreal zones. As one of the most abundant species in a wide range of acidic and mostly nutrient-poor microsites in the United Kingdom, the species displays remarkable variation in morphology, from exuberant patches more than 10 cm across of green-grey frilly lobes 2–3 cm long on shady trees to small, dark brown rosettes of lobes up to only 1 cm on old fenceposts or mountain rocks. The basis of this remarkable morphological diversity and microhabitat tolerance is not understood. Although genetic data suggest that the European material is comprised of two lineages, these appear not to be differentiated either biogeographically or morphologically ( Asher *et al.*, 2023).

Most lichens, including *P. glauca*, form perennial structures and comprise complex systems of at least two and often more co-occurring and symbiotic eukaryotes ( Grimm *et al.*, 2021), though few species have been fully explored using metagenomics. *P. glauca* is comprised of both the dominant Lecanoralean ascomycete fungus and a green alga in the *Trebouxiophyceae* as a symbiotic carbon source. In addition, at least two Basidiomycete microfungi are also known to grow on or within it: the genus-specific parasite *Tremella coppinsii* ( Diederich & Marson, 1988) forms small pink galls and lesions, and the microscopic and asymptomatic cystobasidiomycete yeasts, which are found inconsistently in PCR or metagenomic surveys of the species ( Lendemer *et al.*, 2019; Spribille *et al.*, 2016). The complex biochemistry of lichen fungi includes many bioactive compounds ( Llewellyn *et al.*, 2023); *P. glauca* has been used as a spice and flavour enhancer and may have antimicrobial properties ( Zhao *et al.*, 2021).

The genome of *Platismatia glauca* was sequenced as part of the Darwin Tree of Life Project, a collaborative effort to sequence all named eukaryotic species in the Atlantic Archipelago of Britain and Ireland. Here we present a chromosomally complete genome sequence for *Platismatia glauca*, based on one collection from twigs in the arboretum at Benmore Botanic Garden, Dunoon, Argyll.

## Genome sequence report

**Figure 1.**
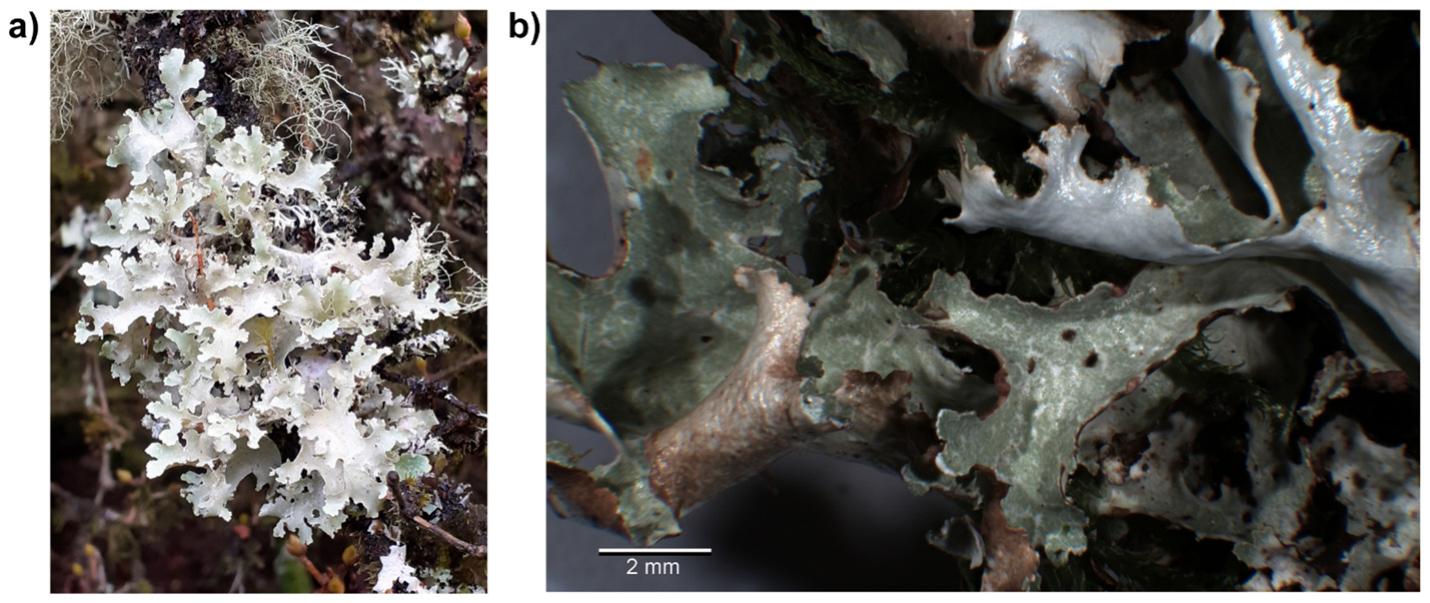
**a**) Photograph of *Platismatia glauca*. **b**) Photograph of the sequenced specimen (EDTOL00467; glPlaGlau9) used for genome sequencing, with 2 mm scale bar.

The genome was sequenced from a specimen R. Yahr 6396 (E) of *Platismatia glauca* ( Figure 1) collected from Benmore Botanic Garden, Argyllshire, UK. (56.02, –4.99). A total of 574-fold coverage in Pacific Biosciences single-molecule HiFi long reads was generated. Primary assembly contigs were scaffolded with chromosome conformation Hi-C data. Manual assembly curation corrected 6 missing joins or mis-joins and removed 5 haplotypic duplications, reducing the assembly length by 1.72% and the scaffold number by 8.33%.

**Figure 2.**
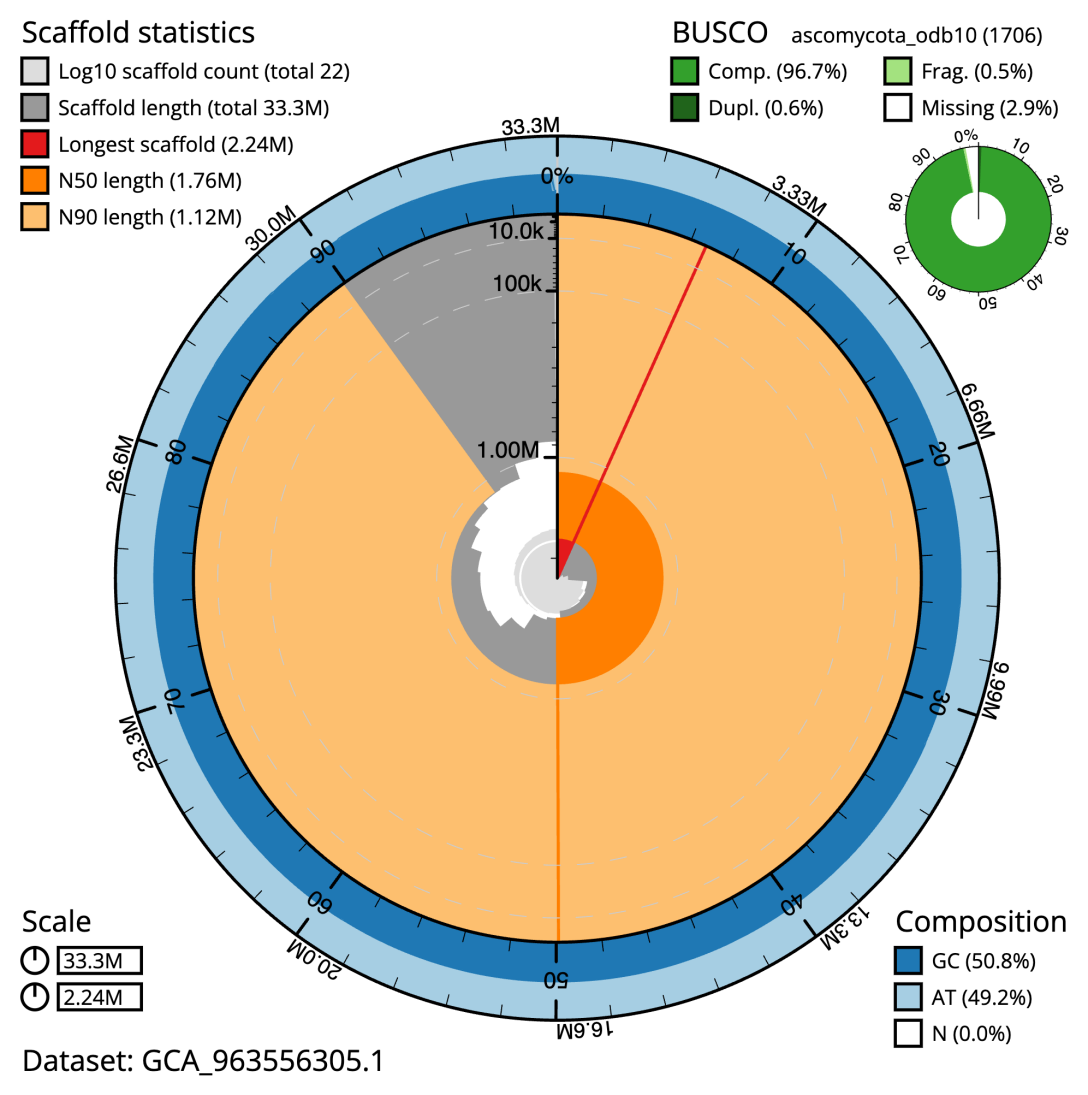
Genome assembly of *Platismatia glauca*, glPlaGlau9.1: metrics. The BlobToolKit snail plot shows N50 metrics and BUSCO gene completeness. The main plot is divided into 1,000 size-ordered bins around the circumference with each bin representing 0.1% of the 33,285,102 bp assembly. The distribution of scaffold lengths is shown in dark grey with the plot radius scaled to the longest scaffold present in the assembly (2,237,538 bp, shown in red). Orange and pale-orange arcs show the N50 and N90 scaffold lengths (1,760,456 and 1,122,404 bp), respectively. The pale grey spiral shows the cumulative scaffold count on a log scale with white scale lines showing successive orders of magnitude. The blue and pale-blue area around the outside of the plot shows the distribution of GC, AT and N percentages in the same bins as the inner plot. A summary of complete, fragmented, duplicated and missing BUSCO genes in the ascomycota_odb10 set is shown in the top right. An interactive version of this figure is available at https://blobtoolkit.genomehubs.org/view/Platismatia_glauca/dataset/GCA_963556305.1/snail.

**Figure 3.**
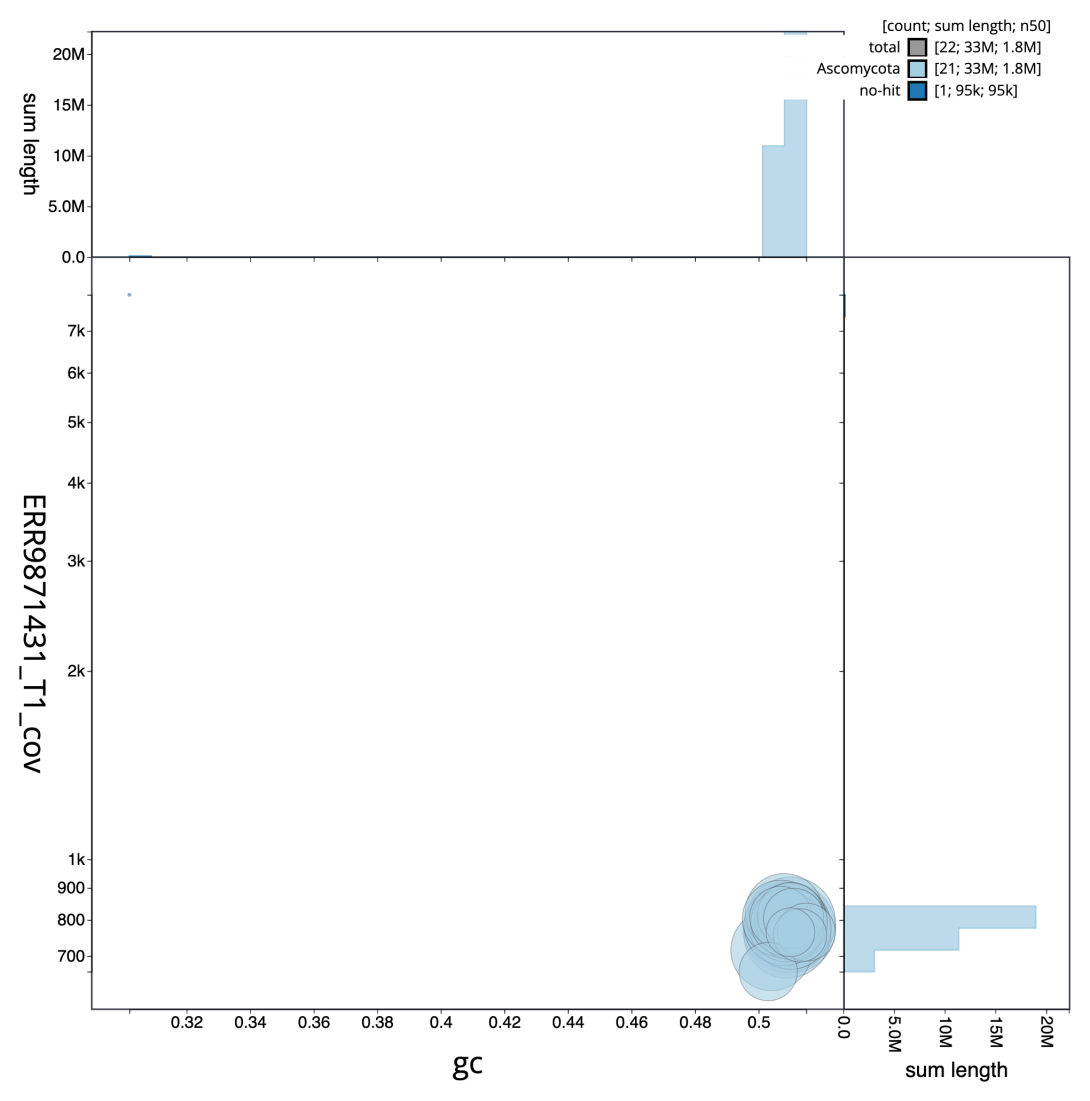
Genome assembly of *Platismatia glauca*, glPlaGlau9.1: BlobToolKit GC-coverage plot. Sequences are coloured by phylum. Circles are sized in proportion to sequence length. Histograms show the distribution of sequence length sum along each axis. An interactive version of this figure is available at https://blobtoolkit.genomehubs.org/view/Platismatia_glauca/dataset/GCA_963556305.1/blob.

**Figure 4.**
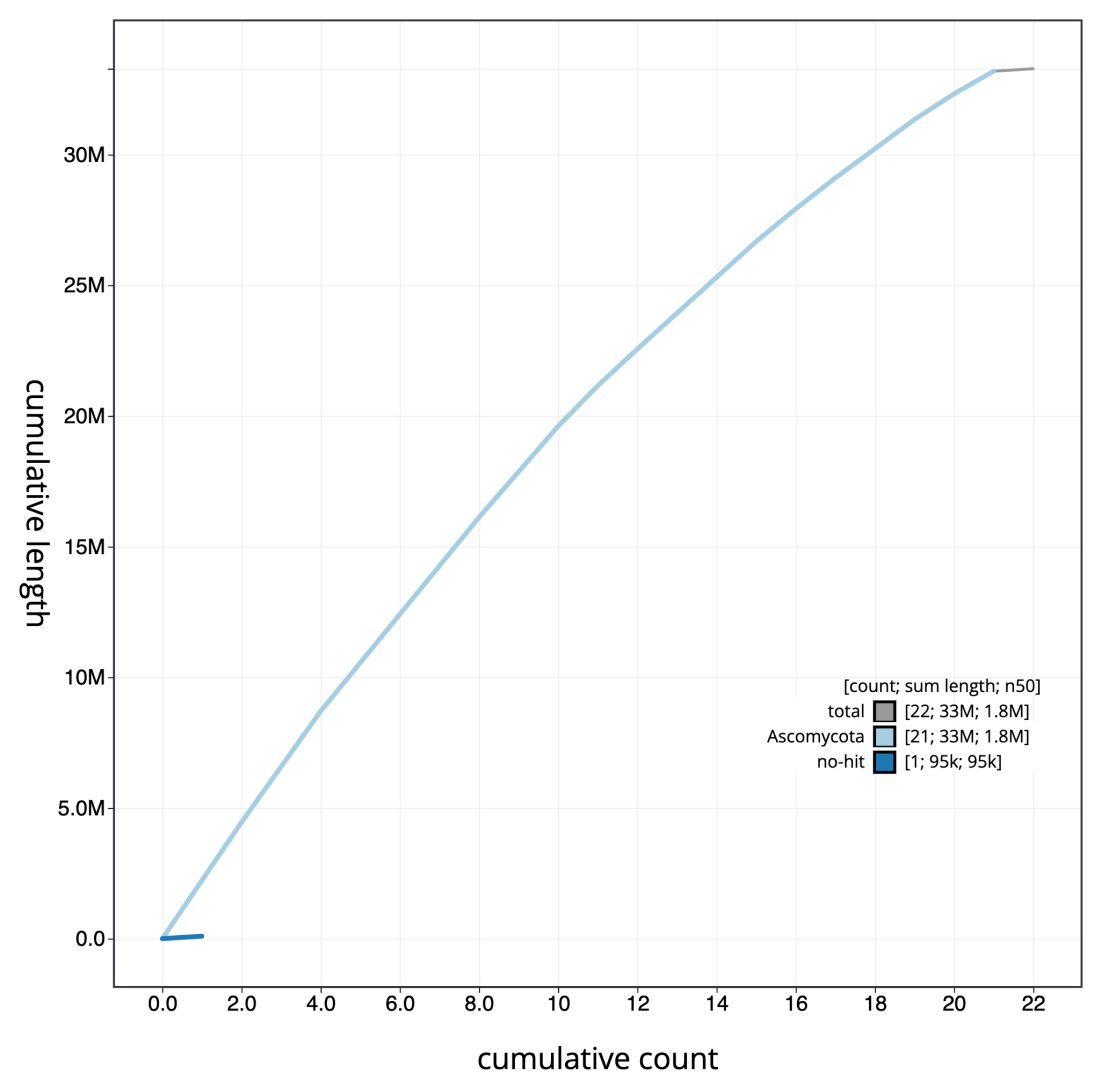
Genome assembly of *Platismatia glauca*, glPlaGlau9.1: BlobToolKit cumulative sequence plot. The grey line shows cumulative length for all sequences. Coloured lines show cumulative lengths of sequences assigned to each phylum using the buscogenes taxrule. An interactive version of this figure is available at https://blobtoolkit.genomehubs.org/view/Platismatia_glauca/dataset/GCA_963556305.1/cumulative.

**Figure 5.**
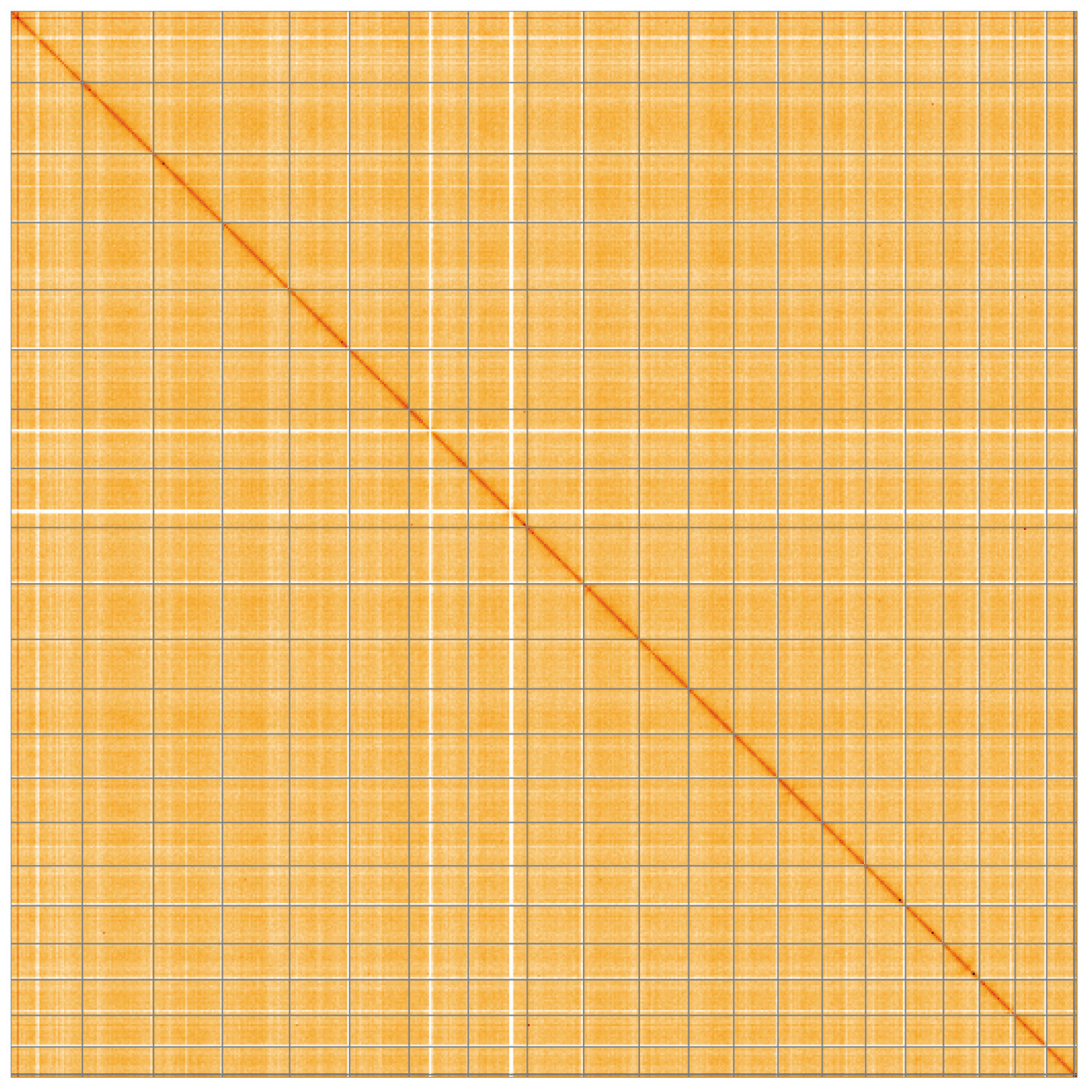
Genome assembly of *Platismatia glauca*, glPlaGlau9.1: Hi-C contact map of the glPlaGlau9.1 assembly, visualised using HiGlass. Chromosomes are shown in order of size from left to right and top to bottom. An interactive version of this figure may be viewed at https://genome-note-higlass.tol.sanger.ac.uk/l/?d=Y5k8oDMFSt2v5EJFijhwwA.

The final assembly has a total length of 33.2 Mb in 21 sequence scaffolds with a scaffold N50 of 1.8 Mb ( Table 1). The snail plot in Figure 2 provides a summary of the assembly statistics, while the distribution of assembly scaffolds on GC proportion and coverage is shown in Figure 3. The cumulative assembly plot in Figure 4 shows curves for subsets of scaffolds assigned to different phyla. Most (99.71%) of the assembly sequence was assigned to 21 chromosomal-level scaffolds. Chromosome-scale scaffolds confirmed by the Hi-C data are named in order of size ( Figure 5; Table 2). The Hi-C data for this assembly were derived from a different sample than the sequence data. Therefore the inversion that can be seen in the Hi-C map on chromosome 4 between ~32–202 kilobases represents the sample used for the Hi-C, and not the underlying sequence. There is no evidence for an inversion in the genome sequence at this location provided by reads spanning the suggested break points. While not fully phased, the assembly deposited is of one haplotype. Contigs corresponding to a second haplotype were found, as this was a highly heterozygous sample, and these were deposited as an alternate haplotype. The mitochondrial genome was also assembled and can be found as a contig within the multifasta file of the genome submission.

**Table 1.** Genome data for *Platismatia glauca*, glPlaGlau9.1.

Project accession data		
Assembly identifier	glPlaGlau9.1	
Species	*Platismatia glauca*	
Specimen	glPlaGlau9	
NCBI taxonomy ID	78070	
BioProject	PRJEB53493	
BioSample ID	SAMEA8597068	
Isolate information	glPlaGlau9, thallus (genome sequencing) glPlaGlau5, thallus (Hi-C sequencing)	
Assembly metrics *		*Benchmark*
Consensus quality (QV)	68.1	*≥ 50*
*k*-mer completeness	100.0%	*≥ 95%*
BUSCO **	C:96.6%[S:96.0%,D:0.6%],F:0.5%, M:2.9%,n:1,706	*C ≥ 95%*
Percentage of assembly mapped to chromosomes	99.71%	*≥ 95%*
Organelles	Mitochondrial genome: 95.06 kb	*complete single alleles*
Raw data accessions		
PacificBiosciences Sequel IIe	ERR9854834, ERR9871431	
Hi-C Illumina	ERR11732958	
Genome assembly		
Assembly accession	GCA_963556305.1	
*Accession of alternate haplotype*	GCA_963556265.1	
Span (Mb)	33.2	
Number of contigs	30	
Contig N50 length (Mb)	1.5	
Number of scaffolds	21	
Scaffold N50 length (Mb)	1.8	
Longest scaffold (Mb)	2.24	

* Assembly metric benchmarks are adapted from column VGP-2020 of “Table 1: Proposed standards and metrics for defining genome assembly quality” from Rhie *et al.* (2021). ** BUSCO scores based on the ascomycota_odb10 BUSCO set using version v5.4.3. C = complete [S = single copy, D = duplicated], F = fragmented, M = missing, n = number of orthologues in comparison. A full set of BUSCO scores is available at https://blobtoolkit.genomehubs.org/view/Platismatia_glauca/dataset/GCA_963556305.1/busco.

**Table 2.** Chromosomal pseudomolecules in the genome assembly of *Platismatia glauca*, glPlaGlau9.

INSDC accession	Chromosome	Length (Mb)	GC%
OY745763.1	1	2.24	51.0
OY745764.1	2	2.22	51.0
OY745765.1	3	2.15	51.0
OY745766.1	4	2.1	51.0
OY745767.1	5	1.87	50.5
OY745768.1	6	1.86	51.0
OY745769.1	7	1.84	51.0
OY745770.1	8	1.84	51.0
OY745771.1	9	1.76	51.0
OY745772.1	10	1.73	50.5
OY745773.1	11	1.55	51.0
OY745774.1	12	1.4	51.0
OY745775.1	13	1.38	51.0
OY745776.1	14	1.38	51.0
OY745777.1	15	1.35	51.0
OY745778.1	16	1.25	50.5
OY745779.1	17	1.18	51.0
OY745780.1	18	1.12	50.5
OY745781.1	19	1.11	51.5
OY745782.1	20	0.98	51.5
OY745783.1	21	0.87	51.0
OY745784.1	MT	0.1	30.0

The estimated Quality Value (QV) of the final assembly is 68.1 with *k*-mer completeness of 100.0%, and the assembly has a BUSCO v5.3.2 completeness of 96.6% (single = 96.0%, duplicated = 0.6%), using the ascomycota_odb10 reference set ( *n* = 1,706).

Metadata for specimens, BOLD barcode results, spectra estimates, sequencing runs, cobionts and pre-curation assembly statistics are given at https://links.tol.sanger.ac.uk/species/78070.

## Methods

### Sample acquisition and nucleic acid extraction

Specimens of *Platismatia glauca* was collected from a mixed conifer arboretum in mesic floodplain in Benmore Botanic Garden, Argyllshire, UK (latitude 56.02, longitude –4.99) on 2021-04-02. The samples were collected live into paper packets into the field, and then cleaned of bryophytes and subsampled. The specimen R. Yahr 6396 (deposited at E) was collected and identified by Rebecca Yahr (Royal Botanic Garden Edinburgh) and subsamples were preserved in a solution of RNAlater (Sigma-Aldrich). The subsample with ID EDTOL00467 (ToLID glPlaGlau9) was used for genome sequencing, and the subsample with ID EDTOL00463 (ToLID glPlaGlau5) was used for Hi-C sequencing. Subsamples were comprised of multiple lobes growing from the same apparent individual on a single twig.

The workflow for high molecular weight (HMW) DNA extraction at the Wellcome Sanger Institute (WSI) Tree of Life Core Laboratory includes a sequence of core procedures: sample preparation; sample homogenisation, DNA extraction, fragmentation, and clean-up. In sample preparation, the glPlaGlau9 sample was weighed and dissected on dry ice ( Jay *et al.*, 2023).

For sample homogenisation, tissue was cryogenically disrupted using the Covaris cryoPREP ^®^ Automated Dry Pulverizer ( Narváez-Gómez *et al.*, 2023). HMW DNA was extracted using the Manual MagAttract v1 protocol ( Strickland *et al.*, 2023b). DNA was sheared into an average fragment size of 12–20 kb in a Megaruptor 3 system with speed setting 30 ( Todorovic *et al.*, 2023). Sheared DNA was purified by solid-phase reversible immobilisation ( Strickland *et al.*, 2023a): in brief, the method employs AMPure PB beads to eliminate shorter fragments and concentrate the DNA. The concentration of the sheared and purified DNA was assessed using a Nanodrop spectrophotometer and Qubit Fluorometer and Qubit dsDNA High Sensitivity Assay kit. Fragment size distribution was evaluated by running the sample on the FemtoPulse system.

Protocols developed by the WSI Tree of Life laboratory are publicly available on protocols.io ( Denton *et al.*, 2023).

### Sequencing

Pacific Biosciences HiFi circular consensus DNA sequencing libraries were constructed according to the manufacturers’ instructions. DNA sequencing was performed by the Scientific Operations core at the WSI on a Pacific Biosciences Sequel IIe instrument. Hi-C data were also generated from tissue of glPlaGlau5 using the Arima2 kit and sequenced on the Illumina NovaSeq 6000 instrument.

### Genome assembly and curation

Assembly was carried out with Hifiasm ( Cheng *et al.*, 2021) and haplotypic duplication was identified and removed with purge_dups ( Guan *et al.*, 2020). The assembly was then scaffolded with Hi-C data ( Rao *et al.*, 2014) using YaHS ( Zhou *et al.*, 2023). The assembly was checked for contamination and corrected as described previously ( Howe *et al.*, 2021). Manual curation was performed using HiGlass ( Kerpedjiev *et al.*, 2018) and PretextView ( Harry, 2022). The mitochondrial genome was assembled using MBG ( Rautiainen & Marschall, 2021) from PacBio HiFi reads mapping to related genomes. A representative sequence was selected for each from the graph based on read coverage, contig size, and its alignments to the related genomes, then MitoHiFi ( Uliano-Silva *et al.*, 2023) was run on this sequence for circularisation and annotation with MitoFinder ( Allio *et al.*, 2020).

### Final assembly evaluation

The final assembly was post-processed and evaluated with the three Nextflow ( Di Tommaso *et al.*, 2017) DSL2 pipelines “sanger-tol/readmapping” ( Surana *et al.*, 2023a), “sanger-tol/genomenote” ( Surana *et al.*, 2023b), and “sanger-tol/blobtoolkit” ( Muffato *et al.*, 2024). The pipeline sanger-tol/readmapping aligns the Hi-C reads with bwa-mem2 ( Vasimuddin *et al.*, 2019) and combines the alignment files with SAMtools ( Danecek *et al.*, 2021). The sanger-tol/genomenote pipeline transforms the Hi-C alignments into a contact map with BEDTools ( Quinlan & Hall, 2010) and the Cooler tool suite ( Abdennur & Mirny, 2020), which is then visualised with HiGlass ( Kerpedjiev *et al.*, 2018). It also provides statistics about the assembly with the NCBI datasets ( Sayers *et al.*, 2024) report, computes *k*-mer completeness and QV consensus quality values with FastK and MerquryFK, and a completeness assessment with BUSCO ( Manni *et al.*, 2021).

The sanger-tol/blobtoolkit pipeline is a Nextflow port of the previous Snakemake Blobtoolkit pipeline ( Challis *et al.*, 2020). It aligns the PacBio reads with SAMtools and minimap2 ( Li, 2018) and generates coverage tracks for regions of fixed size. In parallel, it queries the GoaT database ( Challis *et al.*, 2023) to identify all matching BUSCO lineages to run BUSCO ( Manni *et al.*, 2021). For the three domain-level BUSCO lineage, the pipeline aligns the BUSCO genes to the Uniprot Reference Proteomes database ( Bateman *et al.*, 2023) with DIAMOND ( Buchfink *et al.*, 2021) blastp. The genome is also split into chunks according to the density of the BUSCO genes from the closest taxonomic lineage, and each chunk is aligned to the Uniprot Reference Proteomes database with DIAMOND blastx. Genome sequences that have no hit are then chunked with seqtk and aligned to the NT database with blastn ( Altschul *et al.*, 1990). All those outputs are combined with the blobtools suite into a blobdir for visualisation.

All three pipelines were developed using the nf-core tooling ( Ewels *et al.*, 2020), use MultiQC ( Ewels *et al.*, 2016), and make extensive use of the Conda package manager, the Bioconda initiative ( Grüning *et al.*, 2018), the Biocontainers infrastructure ( da Veiga Leprevost *et al.*, 2017), and the Docker ( Merkel, 2014) and Singularity ( Kurtzer *et al.*, 2017) containerisation solutions.

Table 3 contains a list of relevant software tool versions and sources.

**Table 3.** Software tools: versions and sources.

Software tool	Version	Source
BEDTools	2.30.0	https://github.com/arq5x/bedtools2
Blast	2.14.0	ftp://ftp.ncbi.nlm.nih.gov/blast/executables/blast+/
BlobToolKit	4.3.7	https://github.com/blobtoolkit/blobtoolkit
BUSCO	5.4.3 and 5.5.0	https://gitlab.com/ezlab/busco
bwa-mem2	2.2.1	https://github.com/bwa-mem2/bwa-mem2
Cooler	0.8.11	https://github.com/open2c/cooler
DIAMOND	2.1.8	https://github.com/bbuchfink/diamond
fasta_windows	0.2.4	https://github.com/tolkit/fasta_windows
FastK	427104ea91c78c3b8b8b49f1a7d6bbeaa869ba1c	https://github.com/thegenemyers/FASTK
GoaT CLI	0.2.5	https://github.com/genomehubs/goat-cli
Hifiasm	0.16.1	https://github.com/chhylp123/hifiasm
HiGlass	44086069ee7d4d3f6f3f0012569789ec138f42b84 aa44357826c0b6753eb28de	https://github.com/higlass/higlass
MBG	3a3c79c24169c8155492057ff7bfa7acc4e3fcd8	https://github.com/maickrau/MBG
MerquryFK	d00d98157618f4e8d1a9190026b19b471055b22e	https://github.com/thegenemyers/MERQURY.FK
MitoHiFi	2.2	https://github.com/marcelauliano/MitoHiFi
MultiQC	1.14, 1.17, and 1.18	https://github.com/MultiQC/MultiQC
NCBI Datasets	15.12.0	https://github.com/ncbi/datasets
Nextflow	23.04.0-5857	https://github.com/nextflow-io/nextflow
PretextView	0.2	https://github.com/wtsi-hpag/PretextView
purge_dups	1.2.3	https://github.com/dfguan/purge_dups
samtools	1.16.1, 1.17, and 1.18	https://github.com/samtools/samtools
sanger-tol/genomenote	1.1.1	https://github.com/sanger-tol/genomenote
sanger-tol/readmapping	1.2.1	https://github.com/sanger-tol/readmapping
Seqtk	1.3	https://github.com/lh3/seqtk
Singularity	3.9.0	https://github.com/sylabs/singularity
YaHS	yahs-1.1.91eebc2	https://github.com/c-zhou/yahs

### Wellcome Sanger Institute – Legal and Governance

The materials that have contributed to this genome note have been supplied by a Darwin Tree of Life Partner. The submission of materials by a Darwin Tree of Life Partner is subject to the **‘Darwin Tree of Life Project Sampling Code of Practice’**, which can be found in full on the Darwin Tree of Life website here. By agreeing with and signing up to the Sampling Code of Practice, the Darwin Tree of Life Partner agrees they will meet the legal and ethical requirements and standards set out within this document in respect of all samples acquired for, and supplied to, the Darwin Tree of Life Project.

Further, the Wellcome Sanger Institute employs a process whereby due diligence is carried out proportionate to the nature of the materials themselves, and the circumstances under which they have been/are to be collected and provided for use. The purpose of this is to address and mitigate any potential legal and/or ethical implications of receipt and use of the materials as part of the research project, and to ensure that in doing so we align with best practice wherever possible. The overarching areas of consideration are:

• Ethical review of provenance and sourcing of the material

• Legality of collection, transfer and use (national and international) 

Each transfer of samples is further undertaken according to a Research Collaboration Agreement or Material Transfer Agreement entered into by the Darwin Tree of Life Partner, Genome Research Limited (operating as the Wellcome Sanger Institute), and in some circumstances other Darwin Tree of Life collaborators.

## Data Availability

European Nucleotide Archive: *Platismatia glauca*. Accession number PRJEB53493; https://identifiers.org/ena.embl/PRJEB53493 ( Wellcome Sanger Institute, 2023). The genome sequence is released openly for reuse. The *Platismatia glauca* genome sequencing initiative is part of the Darwin Tree of Life (DtoL) project. All raw sequence data and the assembly have been deposited in INSDC databases. The genome will be annotated using available RNA-Seq data and presented through the Ensembl pipeline at the European Bioinformatics Institute. Raw data and assembly accession identifiers are reported in Table 1.

## Author information

Members of the Royal Botanic Garden Edinburgh Genome Acquisition Lab are listed here: https://doi.org/10.5281/zenodo.4786682.

Members of the Darwin Tree of Life Barcoding collective are listed here: https://doi.org/10.5281/zenodo.4893703.

Members of the Wellcome Sanger Institute Tree of Life Management, Samples and Laboratory team are listed here: https://doi.org/10.5281/zenodo.10066175.

Members of Wellcome Sanger Institute Scientific Operations: Sequencing Operations are listed here: https://doi.org/10.5281/zenodo.10043364.

Members of the Wellcome Sanger Institute Tree of Life Core Informatics team are listed here: https://doi.org/10.5281/zenodo.10066637.

Members of the Tree of Life Core Informatics collective are listed here: https://doi.org/10.5281/zenodo.5013541.

Members of the Darwin Tree of Life Consortium are listed here: https://doi.org/10.5281/zenodo.4783558.
