# Supplementary material for: The genome sequence of a lichen-forming fungus, Platismatia glauca Linnaeus, 1753
Source: Wellcome Open Res. 2024 Aug 12;9:457. [Version 1] doi: 10.12688/wellcomeopenres.22842.1 (PMC11480706; doi:10.12688/wellcomeopenres.22842.1)
Supplement: Peer review history

# Reviewer report: Reviewer response for version 1

**DOI:** 10.21956/wellcomeopenres.25150.r100974

**Published:** 2024-10-15

**Authors:** Klara Scharnagl

**Affiliations:** 1 University of California Berkeley, Berkeley, California, USA

This Data Note presents the full genome sequence of the lichenized fungus *Platismatia glauca*.

*Platismatia glauca* is a widespread and charismatic lichen and providing a reference genome for this species will be a valuable resource. The article provides a good background to the species and to the methods. There is only one noticed typo in the Methods section, where it says "Specimens of *Platismatia glauca* was" needs to either be singular or changed to "were." I am impressed with how clean the mycobiont genome is - I am wondering if I missed the step where the photobiont and other genomes were removed. I would like the authors to elaborate on that.

 Finally, there is excellent transparency in methods and software used for analysis. It would be useful to include code for the genome assembly steps as well.

Are sufficient details of methods and materials provided to allow replication by others?

Partly

Is the rationale for creating the dataset(s) clearly described?

Yes

Are the datasets clearly presented in a useable and accessible format?

Yes

Are the protocols appropriate and is the work technically sound?

Yes

Reviewer Expertise:

Lichenology, fungal ecology, lichen transcriptomics

I confirm that I have read this submission and believe that I have an appropriate level of expertise to confirm that it is of an acceptable scientific standard.

# Reviewer report: Reviewer response for version 1

**DOI:** 10.21956/wellcomeopenres.25150.r100982

**Published:** 2024-10-10

**Authors:** Garima Singh

**Affiliations:** 1 University of Padua, Padova, Italy

The author presents a highly contiguous, chromosomally complete genome sequence for *Platismatia glauca*, representing a significant advancement over previous assemblies of this lichenized fungus. This high-quality assembly, generated using the latest long-read sequencing technology, encompasses one primary haplotype, along with a second haplotype that has been deposited as an alternate. Additionally, the mitochondrial genome has been assembled. The manuscript provides a comprehensive overview of the methodology and includes appropriate citations wherever necessary. Overall, this assembly highlights a substantial enhancement in the genomic resources available for *P. glauca*.

 I have only a few minor suggestions, which are listed below.

 Sincerely,

 Garima Singh

**Suggestions:**

- In the background rephrase “ *P. glauca* is " ***comprised of"*** both the dominant Lecanoralean…” to either *P. glauca* ***comprises*** both the dominant Lecanoralean Or to *P. glauca* is ***composed of*** both the dominant Lecanoralean... Also, remove **“both”** from the sentence.
- In addition, at least two Basidiomycete microfungi are also known to grow on or within it: *the genus-specific parasite **Tremella coppinsii ( Diederich & Marson, 1988) forms small pink galls and lesions, and the microscopic and asymptomatic cystobasidiomycete yeasts,** which are found inconsistently* in PCR or metagenomic surveys of the species

**Rephrase to: **parasite *Tremella coppinsii* (Diederich & Marson, 1988) **,** which forms small pink galls and lesions, ......

- … The complex biochemistry of lichen fungi includes many bioactive compounds ( Llewellyn *et al.*, 2023 ^1^);
  If the sentence is referring to lichenized fungi in general, then the citation is not appropriate as the cited article explores the metagenomes of Teloschistales lichens to identify putative anthraquinone BGCs, and does not talk about bioactive compounds per se.
  I suggest citing review articles dealing with bioactive metabolites of lichens.

**For instance:**

 Lichens - A promising source of bioactive secondary metabolites. Boustie & Grube 2005, Plant Genetic Resources

 0r Bioactive Secondary Metabolites from Lichens in Plant Metabolites: Methods, Applications and Prospects (2020) by Nayaka & Haridas.

- Here we present a chromosomally complete genome sequence for *Platismatia glauca*, based on one collection from twigs in the arboretum at Benmore Botanic Garden, Dunoon, Argyll.

Not entirely wrong but please consider rephrasing to “ *from a lichen thallus collected from twigs*”

- In the sentence: The genome was sequenced from a specimen R. Yahr 6396 (E) of *Platismatia glauca* 

I would suggest to either say a specimen **,** R. Yahr 6396 (E) **, **or use " **the specimen**"

- In the sentence” …. the assembly has a BUSCO v5.3.2 completeness of 96.6% ( **single** = 96.0%, **duplicated** = 0.6%), using the ascomycota_odb10 reference set ( *n* = 1,706).”

Please change single and double to " **single copy BUSCOs**" and " **duplicate BUSCOs"**

- “ **Specimens** of *Platismatia glauca* **was** collected from”

Either change “Specimens” to “Specimen” or “was” to “were”

- The manuscript mentions slightly different sampling sites at two points

**Last sentence in the section background:**

- “ *Platismatia glauca, based on one collection from twigs in the arboretum at Benmore Botanic Garden, **Dunoon, Argyll*** **.”**

**And First sentence of Methods:**

- “ *Specimens of Platismatia glauca was collected from a mixed conifer arboretum in mesic floodplain in **Benmore Botanic Garden, Argyllshire, UK ***

Please consider using one for consistency.

- Subsamples were **comprised of **multiple lobes growing from the same apparent individual on a single twig.

In general, “of” is not used with comprised. Please use either “consists of” or “comprises”.

- The samples were collected live into paper packets into the field, and then cleaned of bryophytes and subsampled.

It would be helpful to understand how the samples were cleaned, specifically whether they were cleaned manually in the field and also examined under a microscope.

- The workflow for high molecular weight (HMW) DNA extraction at the Wellcome Sanger Institute (WSI) Tree of Life Core Laboratory includes a sequence of core procedures: sample preparation; sample homogenisation, DNA extraction, fragmentation, and clean-up. I **n sample preparation, **the glPlaGlau9 sample was weighed and dissected on dry ice

**Please rephrase to: **

- For sample preparation….

Are sufficient details of methods and materials provided to allow replication by others?

Yes

Is the rationale for creating the dataset(s) clearly described?

Yes

Are the datasets clearly presented in a useable and accessible format?

Yes

Are the protocols appropriate and is the work technically sound?

Yes

Reviewer Expertise:

(meta) Genomics, omics, fungi, lichens, secondary metabolites, genome mining, biosynthetic genes, phylogenomics

I confirm that I have read this submission and believe that I have an appropriate level of expertise to confirm that it is of an acceptable scientific standard.

## References

[1] : Metagenomics Shines Light on the Evolution of “Sunscreen” Pigment Metabolism in theTeloschistales (Lichen-Forming Ascomycota). *Genome Biology and Evolution* .2023;15(2) : 10.1093/gbe/evad002 10.1093/gbe/evad002 PMC990750436634008
